# The neural architecture of age-related dual-task interferences

**DOI:** 10.3389/fnagi.2014.00193

**Published:** 2014-07-31

**Authors:** Witold X. Chmielewski, Ali Yildiz, Christian Beste

**Affiliations:** ^1^Cognitive Neurophysiology, Department of Child and Adolescent Psychiatry, Faculty of Medicine, TU DresdenDresden, Germany; ^2^Department of Biopsychology, Institute of Cognitive Neuroscience, Ruhr-University BochumBochum, Germany

**Keywords:** aging, dual-tasking, psychological refractory period (PRP), response selection, fMRI

## Abstract

In daily life elderly adults exhibit deficits when dual-tasking is involved. So far these deficits have been verified on a behavioral level in dual-tasking. Yet, the neuronal architecture of these deficits in aging still remains to be explored especially when late-middle aged individuals around 60 years of age are concerned. Neuroimaging studies in young participants concerning dual-tasking were, among others, related to activity in middle frontal (MFG) and superior frontal gyrus (SFG) and the anterior insula (AI). According to the frontal lobe hypothesis of aging, alterations in these frontal regions (i.e., SFG and MFG) might be responsible for cognitive deficits. We measured brain activity using fMRI, while examining age-dependent variations in dual-tasking by utilizing the PRP (psychological refractory period) test. Behavioral data showed an increasing PRP effect in late-middle aged adults. The results suggest the age-related deteriorated performance in dual-tasking, especially in conditions of risen complexity. These effects are related to changes in networks involving the AI, the SFG and the MFG. The results suggest that different cognitive subprocesses are affected that mediate the observed dual-tasking problems in late-middle aged individuals.

## Introduction

Research has shown elderly and already late-middle aged adults (55 till 65 years of age) to exhibit a vast amount of cognitive deficits concerning challenges of everyday life (Glisky, 2007). An obvious observation is a diminished ability for dual tasking (Allen et al., 2002; Verhaeghen and Cerella, 2002; Verhaeghen et al., 2003; Ivanoff et al., 2009; Hartley et al., 2011) during two or more simultaneously upcoming tasks. Since dual-tasking is required in everyday life, for example in driving a car (Ross et al., 2012), impairments ought to be researched thoroughly in late-middle aged adults due to the impact they have. Up to now deficits in dual-tasking in late-middle aged adults have mostly been verified on a behavioral level (Glass et al., 2000; Verhaeghen and Cerella, 2002; Maquestiaux et al., 2004; Allen et al., 2009) and revealed mixed results. Glass et al. (2000) for example found no decline in the ability processing of two tasks, but increased dual-task time costs, which they assumed to be based on generalized slowing, process-specific slowing, and the use of more cautious task-coordination strategies. Other studies suggested that a decreased ability of task automation (Maquestiaux et al., 2010), or an age-related decline in the control of input processes (Hein and Schubert, 2004), or a decrement in time-sharing at the response-selection stage of processing (Allen et al., 1998), or increased input and output interferences (Hartley and Little, 1999) underlies deficits in dual-tasking. Increased psychological refractory period interference in elderly vanish as an effect of training, i.e., elderly do not show deficits when sufficiently trained on a task, but Maquestiaux et al. (2004) could not provide evidence for this hypothesis. Later on in another experiment with extensive training (Maquestiaux et al., 2013) they assumed a loss of the ability to automatize novel tasks to be responsible for occurring deficits in elderly. As can be seen, the results on dual-task performance in elderly are rather mixed and deficits are evident even after extensive training suggesting that there are dual-tasking deficits in elderly.

Yet, it is unknown what neurofunctional mechanisms underlie these deficits. An analysis of brain regions underlying possible dual-tasking deficits in late-middle aged participants may also provide hints which cognitive processes underlie deficits in dual-tasking. Since processing speed tasks in general have been suggested to reflect potential biomarkers of cognitive aging (Deary et al., 2010) the question what alteration in functional neuroanatomical networks may underlie dual-tasking deficits gains additional relevance. In this regard it is of special interest to examine late-middle aged individuals to approach the question at what age first signs of dual-tasking deficits are evident.

A means to measure dual-tasking abilities is the PRP test (psychological refractory period) (Telford, 1931; Welford, 1952). In this test two consecutive stimuli with very short stimulus onset asynchronies (SOAs), meaning virtually no distance in time, are presented and lead to the occurrence of the PRP effect. That is the appearance of increased reaction times for the second stimulus, when SOAs are decreasing (Pashler, 1984). While the PRP test does not directly test everyday functions, this test is an established and well-validated tool to examine the neurofunctional processing architecture underlying dual-tasking (Sigman and Dehaene, 2008; Hesselmann et al., 2011).

The underlying neuronal architecture in dual-tasking in the PRP has widely been addressed in experiments without consideration of aging aspects (Herath et al., 2001; Marois et al., 2006; Stelzel et al., 2008). In these studies the superior frontal gyrus (SFG) (Jiang and Kanwisher, 2003; Dux et al., 2006; Spence, 2008), as well as medial and middle frontal gyrus (MFG) (Marois and Ivanoff, 2005; Stelzel et al., 2006; Szameitat et al., 2006; Sigman and Dehaene, 2008) have been identified as major contributing areas, due to their increased activation after the appearance of the second stimulus, especially in short SOA conditions. Since these are brain areas are assumed to be especially vulnerable to aging processes according to the frontal lobe/aging hypothesis (Dempster, 1992; West, 1996; Greenwood, 2000; Braw et al., 2011), specific age-dependent activation differences are to be expected and might underlie deficits in dual-tasking in late-middle aged adults. This is also plausible against a neurobiological background: The SFG and MFG are part of fronto-striatal loops known to undergo extended changes during aging (Buckner, 2004). These loops are strongly modulated by the dopaminergic system (e.g., Nieoullon, 2002), which is also known to undergo massive alterations during healthy aging (Bäckman et al., 2000, 2010) affecting dopamine D1 and D2 receptor density (Volkow et al., 1998; Wang et al., 1998), in dopamine availability (Volkow et al., 2000) and in DA transporter availability (Erixon-Lindroth et al., 2005).

Changes in dopaminergic activity and in the basal ganglia (Frank, 2005; Humphries et al., 2006; Leblois et al., 2006) have been identified as some of the major contributing factors for deficits in dual-tasking (e.g., Beste et al., 2012) and response selection in general (Willemssen et al., 2011). In combination with frontal alterations these impairments might cause neurofunctional deficits in task-order scheduling, i.e., the control of the processing order of two nearly simultaneous tasks (Szameitat et al., 2006), or in response inhibition of action sets (Rushworth et al., 2004; Booth et al., 2005; Ghahremani et al., 2012) necessary to chain different tasks at hand. These rather specific deficits might then in turn constitute age-related changes in dual-tasking as examined with the PRP effect. This experiment was conducted to test the hypothesis that decreased ability to activate brain regions located in the SFG and MFG underlie the deficits in dual-tasking in middle-aged individuals.

## Materials and methods

### Study population

Fourteen (*N* = 14) healthy late-middle aged adults (6 females, mean age = 60.51, *SD* = 3.34) within an age span from 55 to 69 and 14 (*N* = 14) young participants (8 females, mean age = 24.37, *SD* = 2.89) between 21 and 29 years of age took part in the experiment. All participants were right-handed according to the Edinburgh Handedness Inventory (Oldfield, 1971) [late-middle aged: 91 ± 6; young: 92 ± 5; *t*_(27)_ = −0.48; *p* = 0.6] and had no neurological or psychiatric antecedents. Participants were matched with regard to the years of education (years at school). The mean years at school was 10.4 ± 3.4 for the late-middle aged adults and 12.1 ± 3.9 for the young participants. The groups did not differ from each other with respect to years of education [*t*_(27)_ = −1.25; *p* = 0.2]. The IQ of the individuals, as assessed using the Mehrfachwahl-Wortschatz test (MWT-B) (Lehrl, 1995) was 110 ± 10.5 for the late-middle aged adults and 107 ± 7.6 for the young participants. The IQ did therefore not differ between groups [*t*_(27)_ = 0.87; *p* > 0.3]. The MWT-B presents the confronts the subjects with several items each item containing a list of words. One of these words reflects a word with a meaning, the other words do not have a meaning. The subjects are required to mark the word with a meaning. The difficulty of items increases during the tests. The number of correctly identified meaningful words equals the IQ. Written informed consent was obtained from all subjects before participating in the experiment. Subjects received financial compensation for their participation. Before testing in the scanner, the participants were familiarized with the paradigm and trained for approximately 15 min, or until a performance level of 90% correct reactions was reached. This study has been approved by the Ethics committee of the Ruhr-University of Bochum and is in accordance with the Declaration of Helsinki.

### Experimental paradigm

In our study we applied a PRP test to examine dual-tasking interferences. An identical paradigm has been used in previous studies by our group (Beste et al., 2013; Yildiz et al., 2013). In this experiment two different kinds of stimuli (visual [letter] and auditory [tone]) were used. In the “letter task” (S2) the participant had to react to the letters “H” and “O,” which were presented at a visual angle of 1.8° × 2.3° via MRI-compatible eye goggles, while in the “tone task” (S1) two tones at a frequency of 500 and 1300 Hz had to be differentiated. Both stimulus types were presented for 200 ms and required specific reactions, which were to be executed as quickly and accurately as possible. That is, the participant had to push the key underlying the left middle finger for the “H,” while for the “O” a keystroke with the right one was requested. For tones the index fingers were used. For the “low” tone (500 Hz) a key-press with the left index finger had to be performed, while the “high” tone (1300 Hz) required a reaction with the right one. Using this response mapping, there is the possibility that the same responding hand or a different responding hand can be used in a particular trial. The stimulus response mapping was counterbalanced across the hands in the groups. Every subject had to complete 640 trials. At the start of the block a brief written instruction was presented on the screen, again describing the following task and assignment of stimuli and fingers.

Each trial started with the presentation of a fixation cross for 1 s, which was followed by two consecutive stimuli, which were randomly presented in rapid succession, accumulating to a total of 160 presentations per stimuli. In our design a tone stimuli (S1) was always followed by a letter (S2). The interval between the two stimuli, the SOA was altered and randomly switched between 16, 133, 500, or 1000 ms.

To guarantee that two trials, as it might arise with two short consecutive SOAs, would not fall into the same TR (time to repetition), the average inter trial interval (ITI) was approximately 1500 ms, jittered between 1000 and 2000 ms, thus avoiding non-differentiable BOLD-signals and co-linearity in the SPM model.

To avoid response grouping (Miller et al., 2009) participants were instructed to process the stimuli consecutively and not only after the presentation of both. This was controlled by excluding trials for the behavioral and fMRI data analysis in which the two reactions occurred in a time span of 100 ms. Reaction times (RT) were obtained for both stimuli. To ensure the execution of two reactions, the next trial was only commencing after two reactions had been obtained. Since this could elicit extensive RTs, trials with RTs of over 1400 ms were treated as misses and excluded from further analysis, also in SPM. The same procedure was applied for trials with at least one incorrect reaction. They were treated as errors and analyzed separately.

### fMRI data collection and analysis

The presentation and timing of the stimuli, response events and the fMRI synchronization were administered with “Presentation” (Neurobehavioral Systems Inc.). A 3T Philips Scanner with a 32-channel bird-cage head coil was employed to conduct functional magnet resonance imaging. MRI-compatible goggles were used to present visual stimuli. Tones were presented through a MRI-compatible headset. The fMRI datasets were gathered using echo planar imaging sequences (EPI) with 3000 ms “time-to-repetition” (TR) and 35 ms “Time to echo” (TE), Flip angle 90° and FOV 256 × 256. In total 40 oblique slices (oriented toward AC-PC line) were obtained.

SPM5 (Statistical Parametric Mapping, Wellcome Department of Imaging Neuroscience, London, UK) was utilized in Matlab (Mathworks, Natick, MA, USA) for image processing and statistical analysis of the fMRI data. Pre-processing consisted of the steps realignment, normalization and smoothing (8 mm isotropic Gaussian kernel)^1^. Data filtering was realized with a high-pass filter applying a cut-off period of 128 s. Since neuroanatomical differences are possibly evident between late-middle aged and young participants a co-registration procedure was applied to account for possible effects of these structural neuroanatomical differences on the localization of brain activations. However, a VBM analysis using the protocol proposed by Good et al. (2001) was conducted (see also Beste et al., 2008) to examine whether were differences in gray matter density between the groups that may bias the effects. This analysis did not reveal significant differences in gray matter density between the groups at a level of *p* < 0.001 and *k* > 10 voxels.

Subsequently “first level” analyses in SPM5 of the individual data were carried out. To control for motion, the motion parameters obtained in the realignment step were fed into the model as regressors of no interest. BOLD contrast differences (t-contrasts) were measured for every participant, as a function of BOLD signal change in comparison to an implicit baseline (noise level), by modeling the corresponding regressors for every single experimental condition to determine scaled beta weights for the conditional events. Using the canonical hemodynamic response function (HRF), regressors containing onset events were modeled separately against the implicit baseline for every SOA condition (i.e., 16 > / 133 > / 500 > / 1000 > noise). Error trials were summarized independent of SOA in an own category as a regressor of no interest. The same was done for trials with “grouped responses” or premature reactions.

All the acquired contrasts were then entered into the “second level” analysis in SPM5 using a full factorial design with the factors “group” (young vs. old) and “SOA” (16, 133, 500, 1000 ms) after random effects analyses were carried out. The anatomical localizations of brain activations were obtained by matching with standard stereotaxic atlas by Talairach and Tournoux (1988). For all utilized analyses, an individual voxel type I error of *p* < 0.001 (uncorrected significance level) and a cluster magnitude of *k* > 30 adjacent resampled voxels were applied.

To plot signal intensities individual activation maxima of the relevant areas were identified, by defining a sphere with a radius of 4 mm around these regions. For every participant the blood oxygen level-dependent (BOLD) signal intensity (beta weights) during every SOA inside this sphere was assessed. For the interaction effect “group × condition” an ANOVA was run on the basis of these extracted signal intensities in SPSS.

### Statistics

Behavioral data was assessed by utilizing ANOVAs and *post-hoc* tests on response times of the SOA-dependent RT2 using the statistical package for social sciences (SPSS) 20.0.0 (SPSS, Inc., 2009, Chicago, IL). The same procedure was applied for activation intensities of relevant brain areas. If necessary, when sphericity could not be assumed, Greenhouse-Geisser correction was applied and tests were Bonferroni-corrected. To guarantee a normal distribution Kolmogorov-Smirnov tests were conducted, showing that all variables were normal distributed (*p* > 0.30).

## Results

### Behavioral data

**Figure 1:**
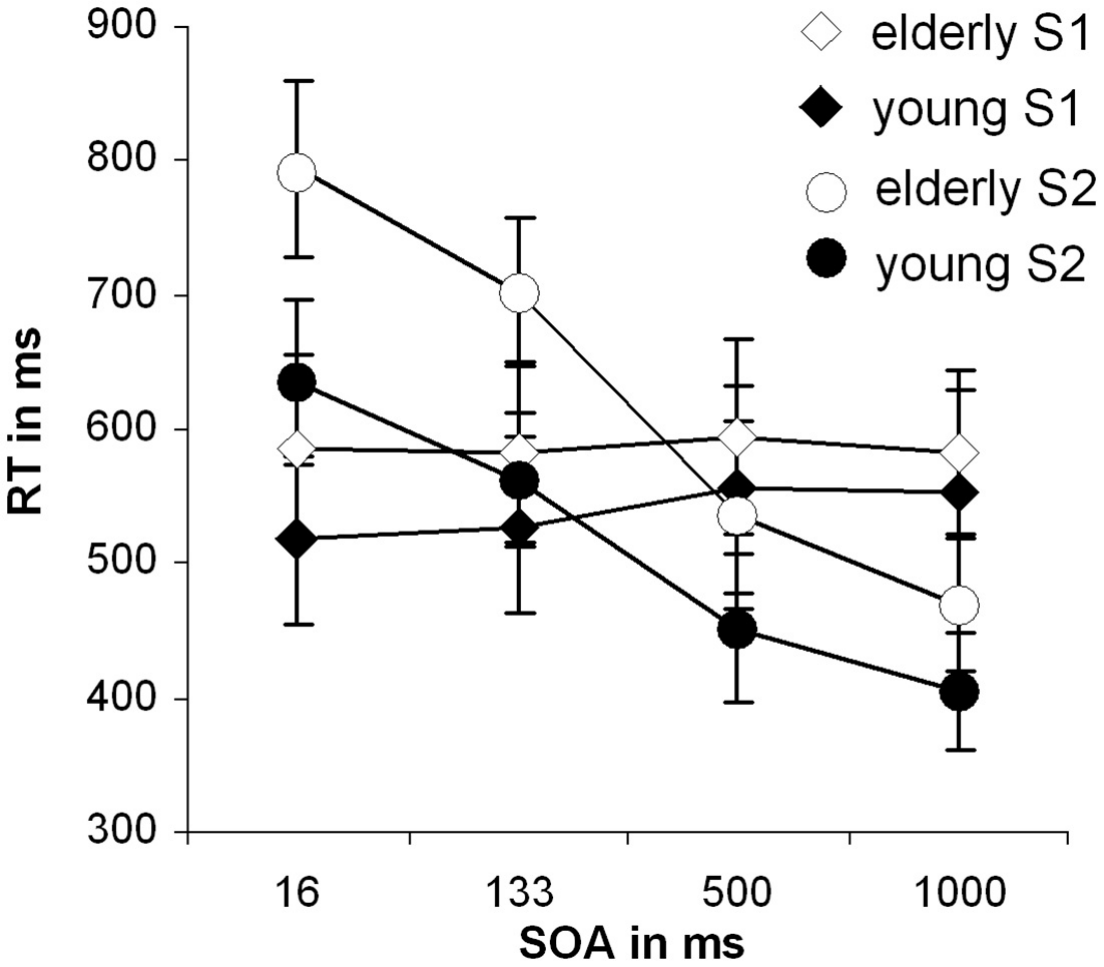
**Behavioral data of the dual-task performance: Mean reaction times with standard deviation of the second stimuli over the different SOA conditions for both groups separately; RT, reaction time; SOA, Stimulus onset asynchrony**.

Figure 1 shows the mean RT and corresponding standard errors of the mean (SEM) for the different SOAs for tones and letters for the late-middle aged and the young group. Tones were always presented first. For the tone stimuli (S1) we found a main effect of SOA length [*F*_(3,81)_= 6.59; *p* = 0.002; η^2^ = 0.196]. Bonferroni-corrected pair-wise comparisons revealed that only SOA 500 differed from SOA 133, (*p* = 0.008) and from SOA 16, (*p* = 0.007). No significant main effect of group could be found [*F*_(1, 27)_ = 3.72; *p* = 0.064; η^2^ = 0.121].

For the second stimulus (S2; letter task) a main effect of SOA length [*F*_(3, 81)_ = 346.16; *p* < 0.001; η^2^ = 0.928] could be observed. Bonferroni-corrected pair-wise comparisons showed that all SOAs differed from each other (*p* < 0.001). This effect resembles the classical PRP effect. Besides that, a significant main effect of group [*F*_(1, 27)_ = 26.04; *p* < 0.001; η^2^ = 0.491] was obtained showing that RTs were slower in the late-middle aged group.

Importantly, also a significant interaction “SOA × group” [*F*_(3, 81)_ = 10.52; *p* = 0.001; η^2^ = 0.280] could be detected. To examine this interaction in more detail we performed Bonferroni-corrected independent samples *t*-tests for each SOA separately. The reaction times differed between the groups in the SOA 16 ms [*t*_(27)_ = −4.87; *p* < 0.001] and 133 ms condition [*t*_(27)_ = −5.01; *p* < 0.001], however, in the SOA 500 and 1000 ms condition there was only a trends toward significance [SOA 500: *t*_(27)_ = −1.64; *p* = 0.060; SOA 1000: *t*_(27)_ = −1.70; *p* = 0.055]. The interaction is therefore driven by the SOA 16 and SOA 133 conditions. We also calculated the slope of the RTs between SOA 16 and SOA 1000 by determining the interval between them. The slope denotes the degree as to which RT2 (i.e., RTs on letters) increase with decreasing SOA length: When comparing the two participating groups concerning their overall SOA-RT2 slopes, a significant difference between the young (−0.34 ± 0.15) and the old group (−0.46 ± 0.16), [*t*_(27)_ = 2.08; *p* = 0.02] could be found. That means, that RT were strongly affected by SOA manipulation in late-middle aged participants. These analyses suggest that there is a real dual-tasking deficit in the late-middle aged group. To ensure validity of this interaction (age and SOA in RT to task 2), a transformation, that corrected for general response latency differences in the two groups was conducted, but evoked no significant alterations in the interaction.

The error rates in the different SOA conditions were analyzed similar to the response times. The mean and standard deviation or error rates in the different SOA conditions and groups are shown in Table 1.

**Table 1:** **Mean and standard deviation of the error rates in the different SOA conditions separated for the different age groups**.

		**Tone task (S1)**	**Letter task (S2)**
Young participants	SOA 16	5.2 (1.8)	6.1 (2.2)
	SOA 133	5.5 (1.2)	5.9 (2.7)
	SOA 500	3.4 (2.1)	6.1 (1.9)
	SOA 1000	4.1 (1.8)	4.9 (2.1)
Late-middle aged	SOA 16	5.8 (2.5)	5.9 (3.1)
	SOA 133	6.3 (3.1)	6.0 (2.8)
	SOA 500	4.2 (2.8)	5.8 (2.1)
	SOA 1000	4.9 (2.9)	4.7 (1.8)

The ANOVAs for the responses on tones and for the responses on letters did not reveal a main effect SOA, or a main effect group, or an interaction effect “SOA × group” (all *F* < 0.9; *p* > 0.4; all η^2^ < 0.01). This shows that the effects were restricted to the speed of processing. Processing accuracy (error rates) was not affected by aging effects, which may also be due to training on the task prior to the scanning session. The results are therefore unbiased with respect to a speed-accuracy trade-off.

### fMRI data

The results of the random effects analyses in the different SOA conditions (16, 133, 500, 1000 ms) separated for the young group and the late-middle aged group are shown in Table 2. The analyses revealed a widely distributed pattern of activation encompassing the caudate nucleus, the SFG, MFG, the anterior and posterior cingulate gyrus, occipital areas, as well as superior temporal areas.

**Table 2:** **Results of the random effects analyses (*p* < 0.001; *k* > 10 voxels)**.

	**Brodmann area**	***x***	***y***	***z***	**Cluster size**
**SOA 16 (LATE-MIDDLE AGED)**					
Caudate nucleus		−14	20	10	104
		−20	−16	28	61
		20	−2	28	32
		10	−22	−30	26
		2	14	16	25
		20	−24	30	21
Cingulate gyrus	BA 31	24	−40	36	11
Anterior cingulate	BA 32	24	32	12	30
Insula	BA13	−42	−28	20	142
		−52	−32	18	32
Parahippocampal gyrus	BA 36	38	−36	−14	11
Fusiform gyrus	BA 37	−40	−48	−8	65
		−38	−36	−6	31
MFG	BA6	−22	−6	40	17
	BA9	−31	13	29	35
	BA10	32	48	20	25
Lingual gyrus	BA 19	30	−66	6	134
**SOA 133 (LATE-MIDDLE AGED)**					
Caudate nucleus		14	26	4	23
		20	−22	30	14
Cingulate gyrus	BA 24	18	8	46	31
	BA 31	22	−46	34	11
		−22	−50	22	11
Insula	BA 13	−42	−36	20	54
		−40	−20	6	11
Parahippocampal gyrus	BA 19	−36	−48	0	15
Superior temporal gyrus	BA 42	−56	−30	14	35
	BA41	−40	−42	8	14
Lentiform nucleus	Putamen	−20	2	20	10
IFG	BA 47	18	32	−14	11
Middle occipital lobe	BA 19	36	−64	8	59
MFG	BA6	−20	−8	41	24
	BA9	−33	12	30	30
	BA10	32	46	22	30
**SOA 500 (LATE-MIDDLE AGED)**					
Caudate nucleus		−20	18	14	33
		16	−36	22	30
		22	−24	30	30
		20	−2	28	11
Cingulate gyrus		−20	−48	22	93
	BA 31	22	−44	36	31
Insula	BA 13	−38	−30	22	51
		38	−6	24	32
		42	−22	22	12
Parahippocampal gyrus	BA 19	−36	−46	−4	24
		−32	−38	8	14
Superior temporal gyrus	BA 22	32	−58	16	96
		68	−14	4	17
		64	−26	4	15
Fusiform gyrus	BA 20	42	−34	−14	15
Superior parietal lobe	BA 7	−18	−44	60	11
MFG	BA6	−19	−9	35	19
	BA9	−33	13	31	35
	BA46	46	44	24	32
**SOA 1000 (LATE-MIDDLE AGED)**					
Caudate nucleus		2	2	16	39
		−32	−38	8	32
		16	−32	24	31
		−22	−14	30	10
Posterior cingulate	BA 23	−10	−32	22	12
Insula	BA 13	−34	−30	22	35
Middle temporal gyrus	BA 39	−30	−58	26	161
MFG	BA6	−18	−8	42	23
	BA9	−32	13	30	29
	BA10	30	44	19	33
**SOA 16 (YOUNG)**					
Caudate nucleus		−14	0	26	11
Anterior cingulate	BA 33	−2	6	20	149
Parahippocampal gyrus	BA 30	−22	−44	10	158
Lentiform nucleus	Putamen	−22	8	2	35
Thalamus	Pulvinar	10	−32	16	282
MFG	BA6	−25	−10	36	22
	BA9	−33	15	29	33
	BA10	30	44	21	35
**SOA 133 (YOUNG)**					
Caudate nucleus		20	−36	16	179
Cingulate gyrus	BA 24	2	−2	22	93
	BA 24	−6	0	22	46
	BA 23	10	−22	22	15
Parahippocampal gyrus	BA 30	−24	−46	6	111
MFG	BA6	−20	−7	41	25
	BA9	−33	17	28	23
	BA46	46	44	30	25
**SOA 500 (YOUNG)**					
Caudate nucleus		20	−38	16	291
		−14	0	26	17
Parahippocampal gyrus	BA30	−20	−44	8	223
MFG	BA6	−22	−6	40	17
	BA9	−35	15	25	40
	BA9	46	30	32	25
**SOA 1000 (YOUNG)**					
Caudate nucleus		20	−40	16	202
		10	−18	24	15
Insula	BA 13	−40	−6	16	18
Parahippocampal gyrus	BA 30	−22	−46	10	167
Lentiform nucleus	Putamen	−28	−10	2	19
MFG	BA6	−21	−7	41	15
	BA9	−34	19	29	35
	BA9	44	31	29	25
Angular gyrus	BA 39	−54	−68	36	13

*The random effects analyses were conducted separately for each SOA condition and age group*.

**Figure 2:**
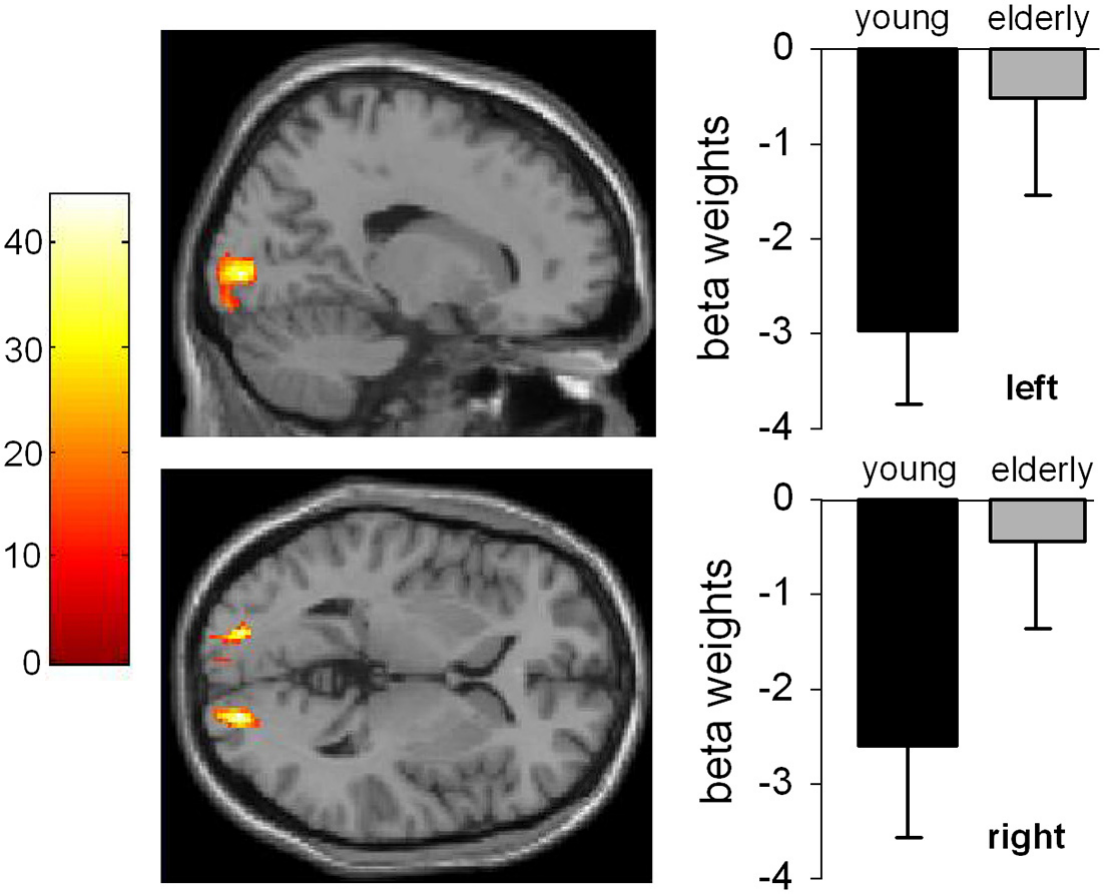
**Main effect of age.** The left part of the Figure shows the activated brain areas (*p* < 0.001; *k* > 30 voxels). The right part of the figure denotes the signal intensities (beta weights) mean and standard deviation.

**Figure 3:**
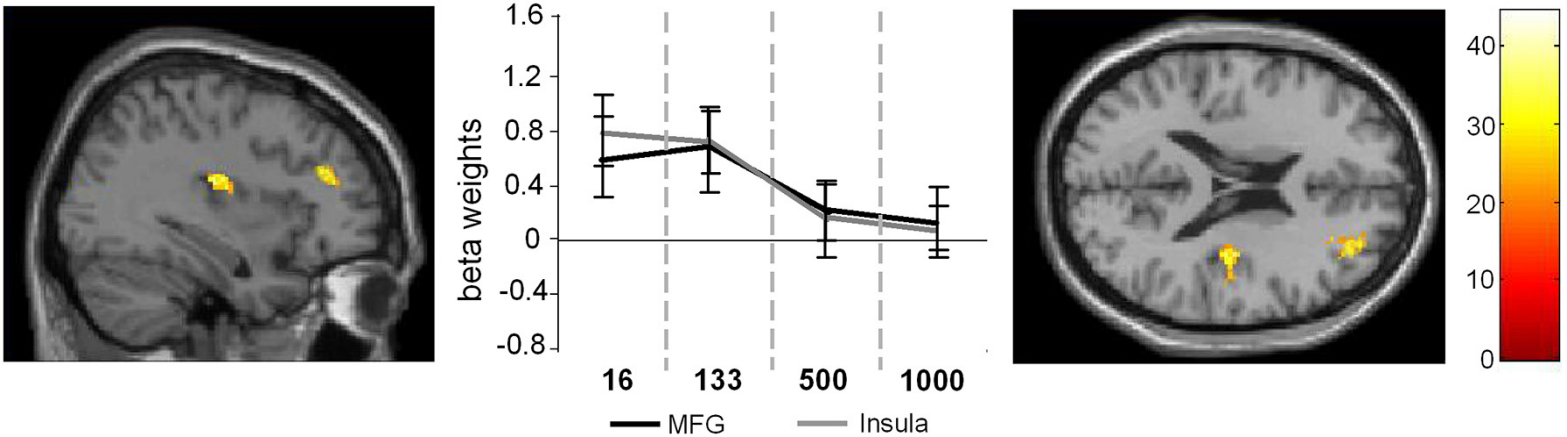
**Main effect of SOA.** The left part of the figure denotes the frontal sections and the coronal view of the activated brain regions (*p* < 0.001; *k* > 30 voxels). The middle part of the figure denotes the signal intensities (beta weights mean and standard deviation) for these regions across the different SOA conditions.

**Figure 4:**
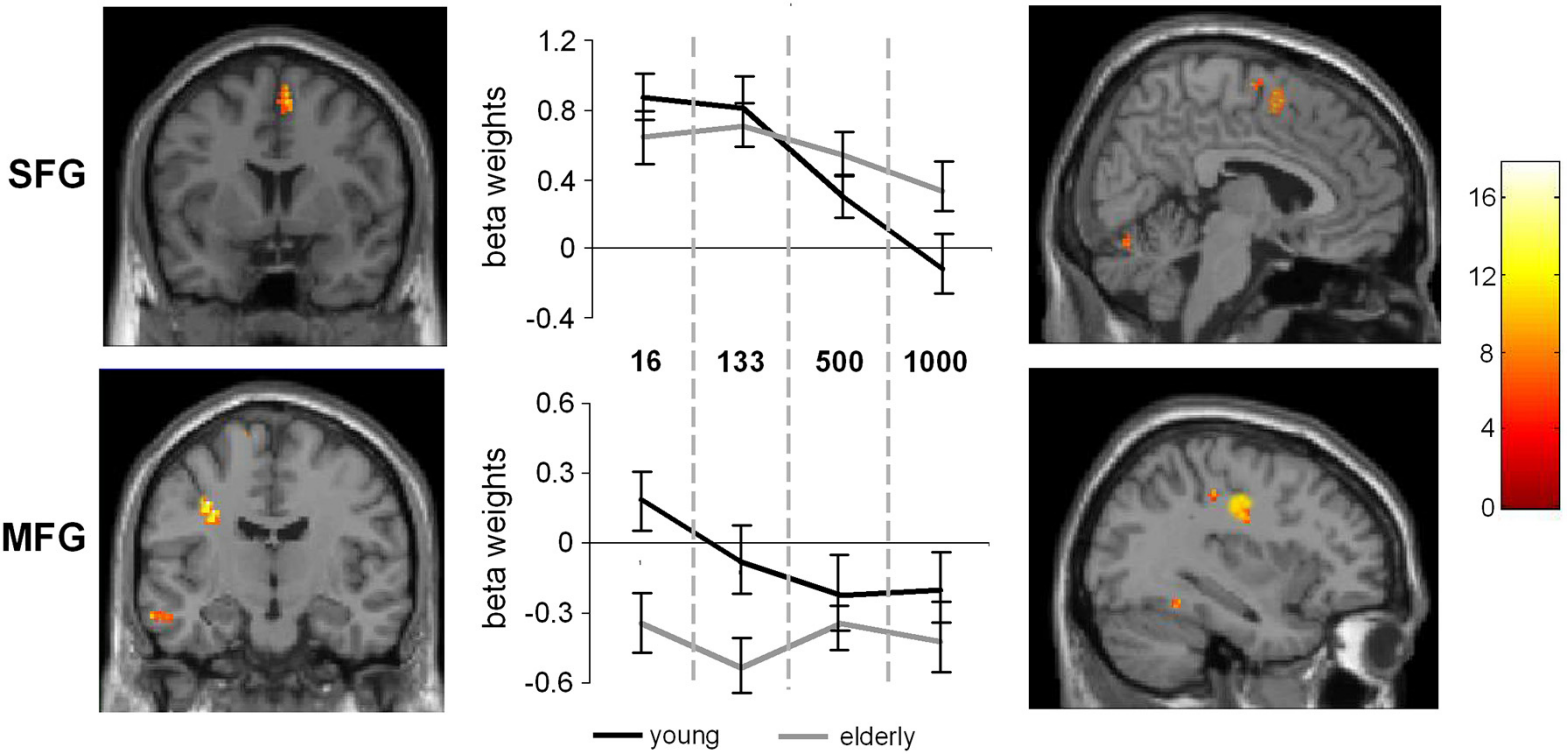
**Interaction of SOA × age.** The left part of the figure denotes the frontal view of the activated regions (SFG and MFG), the right part denotes the sagittal view of the activated brain regions (*p* < 0.001; *k* > 30 voxels). The middle part of the figure denotes the signal intensities (beta weights mean and standard deviation) for the MFG and SFG regions across the different SOA conditions, separated the young and late-middle aged group.

After conducting the random effects analyses the first level contrasts were used in a full-factorial design. For the sake of comparability to the random effects analyses the plots are given using a *p* < 0.001 (uncorrected) and a cluster threshold of *k* > 30 voxels. However, it needs to be noted that the presented results of the full-factorial analyses also withstood an FDR correction at the level of *p* = 0.03 using a cluster threshold of *k* = 10 voxels. The full-factorial design revealed three significant effects: There were main effects of Group (Figure 2), of SOA (Figure 3) and an interaction of Group × SOA (Figure 4).

The main effect of age (Figure 2) revealed significant differences in brain activations in the occipital cortex within the right (18, −90, −3), and the left BA17 (−16, −90, 4), with both higher activations in older subjects.

In the case of the main effect of SOA activation differences could be found in the MFG (32, 48, 20) and in the anterior insula (AI) (36, −14, 16) (Figure 3).

Comparing the activation intensities in these regions using Bonferroni-corrected pair-wise comparisons revealed that for the MFG SOA 16 differed significantly from SOA 500 (*p* = 0.029) and SOA 1000 (*p* < 0.001) and that SOA 133 differed significantly from SOA 500 (*p* = 0.011) and SOA 1000 (*p* = 0.003) [main effect SOA: *F*_(3, 81)_ = 9.76; *p* < 0.001; η^2^ = 0.75].

For the AI the same analysis showed that SOA 16, differed significantly from SOA 500, [*t*_(27)_ = 4.58; *p* < 0.001] and SOA 1000, [*t*_(27)_ = 5.38; *p* < 0.001] and that SOA 133 differed significantly from SOA 500, [*t*_(27)_ = 4.63; *p* = 0.003] and SOA 1000, [*t*_(27)_ = 4.11; *p* = 0.009; main effect SOA: *F*_(3, 81)_ = 10.36; *p* < 0.001; η^2^ = 0.27].

Additionally, the SPM analyses revealed an interaction between SOA and Group (young vs. late-middle aged) in the MFG (BA9) (−30, 12, 30) and the SFG (BA 6) (6, 6, 58). This interaction is shown in Figure 4. Areas overlapping with the MFG location in this interaction were also found for the random effects analyses (refer Table 1).

To examine this interaction in more detail independent samples *t*-tests on the extracted activation intensities were run in PASW. The analyses showed that in the SOA 16 condition the MFG activation in young and late-middle aged participants differed from each other [*t*_(27)_ = 6.63, *p* = 0.001]. Also in the SOA 133 condition, significant differences could be detected between the groups [*t*_(27)_ = 4.87, *p* < 0.001]. In the SOA 500 and 1000 condition, no differences in MFG activation intensities were evident between groups. In the SFG significant differences could also be observed in the SOA 16 condition [*t*_(27)_ = 2.39, *p* = 0.012] and in the SOA 133 condition, [*t*_(27)_ = 2.00, *p* = 0.027], but not for the other conditions^2^.

## Discussion

The intention of the current study was to enlighten the neurofunctional architecture of dual-tasking dysfunctions in late-middle aged adults. As expected, on a behavioral level, late-middle aged participants showed generally increased reaction times in comparison to young subjects. Late-middle aged adults exhibited an even more pronounced PRP effect than younger participants, which was especially due to the SOA 16 and SOA 133 conditions. In comparison to the young group, late-middle aged subjects showed a steeper rise of reaction times, regarding the second stimulus, between the less challenging SOA 1000 and the more demanding SOA 16 condition. However, this rise is mainly determined by the reaction time differences in the shortest, most demanding SOA conditions. These findings are exactly in line with preceding behavioral studies in late-middle aged adults (e.g., Allen et al., 1998; Glass et al., 2000) and confirm our assumptions about age-dependent diminishing of dual-tasking abilities. As error rates were comparable in young and late-middle aged adults, showing that age-related deficits are somewhat restricted to the speed of processing but not to the accuracy of dual-tasking. Furthermore, the finding that performance declines were restricted to the short SOA conditions show that deficits in late-middle aged individuals are restricted to situations in which dual-task processing demands are high. The results on the error rates therefore are not due to a speed-accuracy trade off. The lack of effects in error rates may emerge as a consequence of the training which an accuracy criterion (i.e., more than 90% correct reactions in the training) was utilized. It cannot be excluded that the unaffected accuracy as well as the fact that performance declines in late-middle aged individuals were restricted to the short SOA conditions reflects an effect of the previously conducted familiarization with the task. During training of the PRP task it has been shown that processes at central stages of the response selection bottleneck are altered (Maquestiaux et al., 2004) in a way that training reduces the duration of processing stages, which reduces the size of the PRP effect (Maquestiaux et al., 2004). As the PRP effects is less evident in longer SOA conditions it is possible that previous training lead to a reduction of group difference in the longer SOA conditions.

Concerning fMRI data the following observations could be made. To begin with, the late-middle aged participants showed more activation in the occipital regions than the younger group. This could be due to the attempt to compensate naturally occurring worsening of the visual system (Spear et al., 1994) and attention processes which have been shown to occur in senescence, when processing complex stimuli (Verhaeghen and Cerella, 2002; Commodari and Guarnera, 2008). Since the PRP has been shown to reflect processes of the central response selection bottleneck (Ruthruff et al., 2001; Hazeltine and Ruthruff, 2005) and not an attentional selection bottleneck (Sigman and Dehaene, 2008) we regard this activation related to the visual stimuli of the task used and not as a ground lying part of the network involved in dual-tasking.

Concerning age-related alterations of neurofunctional activation in dependence of task complexity, interactions between group and SOA were found in the SFG and MFG: While late-middle aged participants showed lower activations in the MFG and SFG in the two more complex conditions (i.e., with SOAs 16 and 133 ms), no such differences compared to young subjects could be observed in the less complex conditions with longer SOAs (500 and 1000 ms). The results are therefore well in line with the interaction effect observed for the behavioral data. According to the frontal lobe hypothesis (Greenwood, 2000) the MFG and SFG are especially vulnerable to age-related changes. These changes are of importance for our experimental design, since the MFG is known for its role in action selection (Hazeltine et al., 2003; Karch et al., 2010) and executive control (Koechlin and Jubault, 2006). So far these regions were identified to be important in processes mediating dual-tasking, task switching and response inhibition in paradigms, which involve the coordination of multiple tasks (Dove et al., 2000; Szameitat et al., 2002, 2006; Aron et al., 2004; Talati and Hirsch, 2005; Simmonds et al., 2008). Furthermore, the MFG has been suggested to be involved in detecting response conflicts between target and distractor stimuli (Bunge et al., 2002; Weissman et al., 2002). As alternate explanations, general activity monitoring (Petrides et al., 1993) and selection of appropriate tasks (Rowe et al., 2000) were proposed. All these functional roles of the MFG do not foreclose each other and it is possible that in the short SOA conditions these functions of conflict resolution and the coordination of multiple tasks are attenuated in late-middle aged individuals. According to Szameitat et al. (2006) the MFG is involved in task order scheduling in the PRP task. Opposed to the study by Szameitat et al. (2006) we did not directly manipulate task order scheduling, even though differences in MFG activity are observed. Yet, aspects of task order scheduling in the sense of task switching effects have frequently been discussed to underlie the classical PRP effect especially in short SOA conditions (see Lien and Procotor, 2002; Oriet and Jolicoeur, 2003; Sigman and Dehaene, 2006; Jentzsch et al., 2007), where strongest effects between the groups were observed. It is therefore plausible that late-middle aged people exhibited a greater PRP effect in combination with decreased activation of the MFG in the short SOA conditions, we expect the MFG mediated task-order scheduling deficits, to be partially responsible for the exhibited age-dependent dual-tasking deficits. In the SFG, similar differential activation changes depending on SOA and age group occurred. So far, Paulus et al. (2005) found the SFG to be activated during the response stage of decision making. The observed lower activation of the SFG might therefore be interpreted as a deficit in response selection processes in late-middle aged adults. However, processes related to the inhibition of task sets (Li et al., 2006) and related to switching to another task set (i.e., of the second stimulus/task) have been shown to play a role in dual-tasking (Sigman and Dehaene, 2006) and may therefore also underlie the deficits observed in late-middle aged adults. The observations made for the MFG and SFG may therefore suggest that dual-tasking deficits in late-middle aged adults in conditions with high overlap between the two to be executed tasks may arise as a consequence of a SFG-mediated deficit in response inhibition and a MFG-mediated deficit in task-order scheduling.

The main effect of SOA revealed modulations of BOLD response in the MFG and AI, which are known to be major contributing regions in this phenomenon (e.g., Szameitat et al., 2006). Here, in both groups, decreasing SOAs led to an increased activation of the MFG and the AI. Changes in MFG activations underlines the finding by Szameitat et al. (2006) that also independently of age a processes related to task-order scheduling might be responsible for the general occurrence of the PRP effect. However, the main effect of SOA and the interaction of SOA and group in the MFG are located in different hemispheres, which might alternatively also indicate a decreased efficacy (not activation) of the right MFG in task-order scheduling in late-middle aged participants. This in turn might elicit additional recruitment of the left MFG, which anyways doesn't seem to be able to compensate the occurring dual-tasking deficits in late-middle aged participants. Activations of the AI were reported in many experiments examining response selection and control. Altered activation in the AI was observed as a part of a bigger network in tasks requiring the coordination of action alternatives and interference control (e.g., Jiang and Kanwisher, 2003; Sanfrey et al., 2003; Paulus et al., 2005; Wager et al., 2005; Marois et al., 2006; Swick et al., 2011). Since the PRP-test involves the coordination of two tasks, which cause interference with decreasing SOAs, we assume that the AI's function might lay in inhibiting a response to a stimulus, or an action set, so that target stimuli can be processed efficiently.

As a limitation of the study it needs to be acknowledged that the sample size is restricted and that no other tasks examining executive control functions were examined. The VBM analysis did not reveal differences in frontal areas and the AI, which have been reported previously in elderly (e.g., Matsuda, 2013). However, it is possible that this is due to the fact that the participants examined were only late-middle aged. Together with the restricted sample size this may lead to the lack of effects in gray matter density in these areas. Yet, it needs to acknowledged that other studies found significant differences even in small samples and similar low age (e.g., Whitwell and Jack, 2005). It is possible that this reflects a cohort effect and that some cohorts in late-middle age show differences whereas other do not show differences. This may depend on factors other than examined in the current study.

In summary, we suggest the age-related deteriorated performance in dual-tasking, especially in conditions of risen complexity, already occur in late-middle aged participants (55 till 65 years of age) are may be based on two neurofunctional deficits: First, it might be due to a decreased ability to inhibit succeeding response sets especially in conditions with short SOA (i.e., fast succession of different response sets), which we assume to be mediated by a bigger network including the AI and the SFG. Second, the declined dual-tasking performance in late-middle aged adults might be based on a decreased ability for task-order scheduling in conditions of higher complexity, which we assume to be caused by age-related alterations in the MFG. The results suggest that different processes may contribute to deficits in dual-tasking in late-middle aged individuals and these have distinct neuronal correlates.

## Author contributions

All authors contributed to the conception of the work, data acquisition, analysis, and interpretation of the data. All authors contributed to drafting the work and finally approved the version to be published. The authors agree to be accountable for all aspects of the work in ensuring that questions related to the accuracy or integrity of any part of the work are appropriately investigated and resolved.

### Conflict of interest statement

The authors declare that the research was conducted in the absence of any commercial or financial relationships that could be construed as a potential conflict of interest.

## Footnotes

^1^Analyzing the data using a 6 mm isotropic Gaussian kernel did not change the pattern of results. The results are therefore unbiased with respect to the usage of different kernel sizes (i.e., 6 and 8 mm).

^2^Significances are given using Bonferroni-correction. Applying another correction method (i.e., Tukey HSD) did not change the pattern of results.
